# A Two-Level Clustered Consensus-Based Bundle Algorithm for Dynamic Heterogeneous Multi-UAV Multi-Task Allocation

**DOI:** 10.3390/s25216738

**Published:** 2025-11-04

**Authors:** Yichao Wang, Chunjiang Wang, Shuangyin Ren

**Affiliations:** 1 Department of Systems Engineering, Academy of Military Sciences, Beijing 100000, China; wycchao0406@163.com; 2 School of Systems Engineering, PLA Information Support Force Engineering University, Wuhan 430000, China; wangcj61@sina.com; 3 Institute of Network and Cloud Security, PLA Unit 32008, Beijing 100000, China

**Keywords:** Multi-UAV task allocation, resource heterogeneity, consensus-based bundle algorithm, communication topology clustering, K-medoids clustering

## Abstract

In multi-UAV cooperative tasks, dynamic communication topologies and resource heterogeneity present significant challenges for distributed task allocation, leading to high communication overhead and poor task-resource matching, which in turn increases computational costs. While the Consensus-Based Bundle Algorithm (CBBA) offers a robust decentralized framework, its scalability and adaptability in heterogeneous, large-scale scenarios are limited. To overcome these issues, this paper introduces a novel Two-Level Clustered CBBA (TLC-CBBA). In the first-layer clustering, UAVs are grouped based on communication topology using graph-theoretic centrality measures to rank node importance, followed by clustering based on shortest-path distances to minimize communication costs. In the second-layer clustering, a resource-balanced and distance-aware K-medoids algorithm is applied within each subgroup obtained from the first-layer clustering, taking into account UAV resource heterogeneity and spatial proximity. This method ensures spatial compactness among UAVs within each subgroup while achieving a more balanced distribution of total resources across clusters. Finally, after completing the two-level clustering, each subgroup executes CBBA for local task bundling and consensus, while the cluster centers coordinate inter-cluster communication to guarantee globally consistent and conflict-free task allocation. Simulations across diverse mission scenarios and UAV team sizes demonstrate that TLC-CBBA substantially outperforms CBBA and its variants (DMCHBA, G-CBBA, and Clustering-CBBA) in terms of communication efficiency, total task score, runtime, and significance analysis. The proposed TLC-CBBA demonstrates strong robustness and scalability for heterogeneous multi-UAV task allocation in dynamic environments.

## 1. Introduction

The rapid advancement of UAV technology has endowed these systems with exceptional mobility, self-organizing capabilities, and rapid responsiveness, enabling their widespread deployment across a variety of civilian sectors, including transportation, communication relays, and disaster response. UAVs also play a critical role in military applications, such as battlefield reconnaissance, precision strikes, and electronic suppression [1,2,3]. In highly dynamic and constraint-driven operational environments, multi-UAV systems show remarkable potential for executing complex missions and achieving scalable swarm coordination, owing to their inherent flexibility and distributed autonomy [4,5]. Within such systems, task allocation plays a critical role, as it directly affects coordination efficiency, communication overhead, and overall mission success. Consequently, it has emerged as a central research focus in both multi-agent systems and contemporary aerospace operations [6].

As UAV systems expand in scale and task complexity grows, task allocation has become a fundamental challenge in mission execution [7]. Efficient task allocation not only accelerates task completion and improves accuracy but also reduces resource consumption and communication load. However, task allocation presents significant problems, as it involves matching a diverse range of UAV platforms with various task objectives, each constrained by factors such as platform capabilities, task priorities, and stringent timelines [8]. Therefore, developing a task allocation algorithm that is both efficient and capable of ensuring system stability in complex, dynamic environments has emerged as a central research challenge in the field of multi-UAV systems.

Multi-UAV task allocation methods are primarily categorized into centralized and distributed types [9]. Centralized methods rely on a central node for task allocation, making them suitable for small-scale systems. Nevertheless, they suffer from long computation times and poor robustness when executing dynamic tasks in large-scale swarms [10]. These methods typically model task allocation as combinatorial optimization problems, such as the Multiple Traveling Salesman Problem (mTSP) [11], the vehicle routing problem (VRP) [12], the multiple traveling salesman problem (MTSP) [13,14] mixed integer linear programming (MILP) [15], the dynamic network flow optimization (DNFO) [16], and solve them using metaheuristic algorithms [17]. While they perform well in small-scale static scenarios, their heavy reliance on a central scheduler results in poor fault tolerance, high communication overhead, and difficulty adapting to communication-constrained or node-vulnerable high-intensity combat environments. In contrast, distributed methods offer a more flexible solution, where UAVs collaborate equally and complete task allocation through communication and negotiation. This enhances the robustness and dynamic responsiveness of the system, although it typically requires more real-time communication.

In current research, distributed algorithms have attracted significant attention due to their suitability for large-scale dynamic systems. These algorithms typically optimize resource allocation while adhering to task constraints through iterative optimization. While these methods are widely adopted, contract-net methods [18] and market-based approaches [19] may struggle with efficiency and stability in dynamic environments. Algorithms based on Markov decision processes [20] and model predictive control [21] can achieve global coordination, but are often hindered by long convergence times. To overcome these limitations, the Consensus-Based Bundle Algorithm (CBBA) has become a widely adopted distributed task allocation algorithm, offering enhanced scalability and robustness due to its decentralized nature. CBBA enables rapid and consistent task allocation through local task bundling, reward evaluation, and distributed consensus processes, making it especially well-suited for applications where targets frequently change or communication topologies are highly dynamic [10,22,23].

Although CBBA and its variants alleviate some of the issues related to distributed communication and collaborative allocation, they still face two major challenges in multi-agent collaborative formations and heterogeneous task-resource matching: On the one hand, as the number of agents and task complexity increase, redundant communication and conflicting information exchanges significantly burden the communication system, delaying convergence [24]; on the other hand, traditional CBBA assumes uniform performance across UAV platforms, overlooking heterogeneity in payload capacity, range, sensor modules, and other aspects [25], which weakens the ability to match tasks with resources, thereby affecting task success rates and platform resource utilization efficiency. Furthermore, resource allocation imbalance is also a significant issue [26]. Ignoring differences between platforms, some UAVs may become overloaded while others remain underutilized, resulting in inefficient resource utilization, increased failure risks, and ultimately impacting task efficiency. Therefore, balancing resource allocation while minimizing communication overhead has become a key research direction in multi-UAV systems.

To address the problems posed by dynamic communication topologies and resource heterogeneity in multi-UAV collaborative tasks, this paper proposes a novel Two-Level CBBA (TLC-CBBA). In the first clustering layer, UAVs are grouped based on communication topology and graph-theoretic centrality measures to optimize node importance, followed by clustering based on shortest path distances to minimize communication costs. In the second clustering layer, K-medoids clustering is used to further optimize each communication cluster, taking resource heterogeneity and spatial proximity into account, thereby enhancing intra-cluster coordination and task compatibility. The CBBA performs local task bundling and consensus within each cluster, while lightweight inter-cluster coordination ensures global, conflict-free task allocation. Simulation results demonstrate that TLC-CBBA significantly outperforms CBBA and its variants (such as DMCHBA, G-CBBA, and Clustering-CBBA) in communication efficiency, task reward, and execution time. The proposed algorithm shows the strong robustness and scalability in dynamic environments, making it well-suited for heterogeneous multi-UAV task allocation.

The main contributions of this paper are as follows:

- A novel TLC-CBBA integrates hierarchical clustering with CBBA for efficient task allocation. The first layer groups UAVs using graph-based centrality to reduce communication cost, while the second layer employs a resource-balanced, distance-aware K-medoids algorithm to enhance coordination and compatibility. CBBA operates within each sub-cluster, and cluster heads coordinate globally to achieve conflict-free allocation.
- A communication network-based grouping strategy is introduced as the first clustering layer. It leverages graph-theoretic centrality to cluster UAVs and assign group heads, enabling inter-group communication through these nodes while optimizing the network topology and mitigating sparsity and redundancy.
- A resource-balanced and distance-aware K-medoids clustering strategy is designed for the second clustering layer. It refines first-layer sub-clusters by considering UAV resource features and spatial proximity, achieving balanced resources and compact clusters. Central nodes act as communication hubs to enhance coordination, reduce redundancy, and improve task–resource efficiency.

The remainder of the paper is structured as follows. Section 2 provides a detailed review of related work; Section 3 presents the theoretical foundation of the proposed method; Section 4 describes the detailed process of the proposed method; Section 5 presents the experimental results and discussion; and finally, Section 6 concludes the paper and outlines future research directions.

## 2. Related Work

Task allocation aims to efficiently assign tasks to the most suitable UAVs, ensuring task completion in the shortest time, which makes it a combinatorial optimization problem [27]. Despite a limited number of UAVs and tasks, multi-UAV task allocation remains NP-hard. To address this, several approximation algorithms have been developed to provide efficient solutions. Task allocation methods are generally divided into centralized and decentralized categories. Centralized approaches mainly include optimization techniques such as integer programming [28], graph theory [29], and exhaustive search, which are suitable for small UAV swarms, and metaheuristic algorithms like genetic algorithms [30], particle swarm optimization algorithm [31], ant colony optimization algorithm [32], and hybrid breeding optimization algorithm [33]. These methods have been shown to identify local or global optimal solutions within a given timeframe. Centralized approaches, however, rely on a central controller to generate and distribute task plans. While they provide global optimal solutions, they come with high computational and communication costs and are less robust, especially when there are single points of failure or issues in communication dynamics. Unlike centralized approaches, decentralized task allocation algorithms—such as those based on game theory [34], auction algorithms [35], and consensus algorithms [36]—eliminate the need for central control. Each UAV independently generates its task list and resolves conflicts through communication with neighbors, enhancing efficiency, robustness, and reducing the risk of single points of failure.

In addition to task allocation, distributed cooperation and motion planning have also been extensively studied in multi-UAV systems. For example, Jin et al. [37] proposed a formation-based cooperative source-seeking algorithm that uses consensus filters and a gradient-free optimization method to enable quadrotor UAVs to collectively locate a source under limited communication. This method effectively addresses the challenges of gradient estimation and robustness in maintaining formation during source localization. Zhou et al. [38] developed a spatial–temporal joint trajectory optimization planner that allows palm-sized UAV swarms to autonomously navigate in highly cluttered and unknown environments, solving key issues such as obstacle avoidance, inter-UAV collision prevention, and swarm coordination.

Among decentralized task allocation methods, auction-based approaches, especially those utilizing market mechanisms, are preferred for their low computational complexity and high operational efficiency, making them well-suited for decentralized environments [36]. Choi et al. [35] combined auction mechanisms with consensus protocols, introducing the CBBA (Consensus-Based Bundle Algorithm). Savkin et al. [39] proposed an efficient navigation algorithm and proved its asymptotic optimality. Specifically, as the area of the region approaches infinity, the ratio of the algorithm’s revisit period to the minimum possible revisit period converges to one. Unlike traditional auction algorithms, CBBA eliminates the need for an intermediary auctioneer, with each UAV following consistent bidding rules. Studies have demonstrated that CBBA can converge to at least 50% of Nash equilibrium solutions within a relatively short period [40]. Achieving consensus requires significant information exchange, resulting in high communication costs. Team CBBA (T-CBBA) [41] accommodates large-scale, complex tasks by pre-assigning operators to teams. It lacks a clear method for selecting a core communication node within a cluster, and its effectiveness across different network structures remains unproven. The Cluster-formed CBBA (CF-CBBA) [42] employs parallel clusters to reduce communication and achieve conflict-free task assignments. Johnson et al. [43] proposed asynchronous rules to resolve task conflicts, reducing unnecessary communication and minimizing overhead in CBBA’s asynchronous system. Fu et al. [44] divided robots into groups, where each group generated and shared its task plan with others through a two-level consensus rule to handle large-scale problems. The cluster-first strategy [45] tackles task allocation in search and rescue scenarios by implementing consensus-driven algorithms that assign tasks to robots with similar functions, thus improving scheduling efficiency. Building on CBBA, Kim et al. [46] introduced the decentralized greedy task allocation algorithm (MCDGA), which reduces communication by requiring UAVs to discard irrelevant bids and minimize overhead. Task reassignment, however, presents challenges and can lead to resource wastage. Grouped CBBA (G-CBBA) [8] groups UAVs based on task preferences, minimizing unnecessary bid distribution to enhance communication efficiency while maintaining the CBBA framework. In environments with limited communication, where target allocation is random and inter-UAV communication with similar bidding intentions is not guaranteed, this grouping strategy may not always yield the desired results.

Designing more complex tasks enables more accurate simulations of real-world scenarios, requiring precise task selection and advanced decision-making. Multi-UAV tasks demand coordinated efforts, particularly for those involving specialized equipment. In incomplete connectivity scenarios, Nayak et al. [47] evaluated asynchronous CBBA’s performance. While CBBA provides a distributed framework, it struggles with complex tasks. To address this, CBBA-TCC, an extension with task coupling constraints, was proposed for search and rescue scenarios [9]. It adds an external consistency phase to handle multi-UAV allocation involving heterogeneity and coupling constraints. Wang et al. [48] introduced the Consensus-Based Timetable Algorithm (CBTA) to address task allocation in decentralized multi-UAV systems, aiming to minimize the start time of each task and, indirectly, the average start time for all tasks.

Zhao et al. [49] developed a heuristic Performance Impact (PI) algorithm to enable parallel iterations on UAVs with local communication, assessing and updating tasks. Whitebook et al. [50] enhanced exploration by adding a softmax operation to overcome the local optimum issue in PI. Turner [51] proposed the PI-MaxAss algorithm, which maximizes task assignments by creating time slots for unassigned tasks. Yang et al. [52] tackled task reassignment in dynamic multi-UAV systems using a distributed framework, employing sub-team formation and partial task release for conflict-free reallocation with minimal data exchange. Wang et al. [53] introduced the EEPI algorithm, which maximizes task execution success without the need for rescheduling.

Although the aforementioned methods have advanced multi-UAV task allocation, they still encounter critical problems in dynamic environments, including excessive communication overhead in dynamic networks, insufficient consideration of resource heterogeneity, and limited scalability in complex task-coupling scenarios. Such shortcomings often lead to inefficient resource utilization and reduced task success rates. To address these limitations, we propose a Two-Level Clustered CBBA (TLC-CBBA) (see Section 4), which systematically integrates clustering with CBBA to enhance communication efficiency, task performance, execution time, heterogeneity handling, and scalability.

## 3. Task Allocation Problem

### 3.1. Problem Statement

This paper investigates decentralized task allocation in multi-UAV systems where heterogeneous UAVs, constrained by task types and time windows, possess different resources and thus can execute only specific tasks. We consider a system consisting of $N_{u}$ UAVs categorized into two types, as shown in Equation (1).

(1) $$U=\left\{U_{1},U_{2},\ldots ,U_{N_{u}}\right\}=\left\{U_{1}^{A},U_{2}^{A},\ldots ,U_{N_{A}}^{A},U_{N_{A+1}}^{B},U_{N_{A+2}}^{B},\ldots ,U_{N_{A}+N_{B}}^{B}\right\},$$

where $N_{A}$ is the number of UAVs of type A, $N_{B}$ is the number of UAVs of type B, and $N_{u}$ is the total number of UAVs.

The task set is defined as in Equation (2).

(2) $$T=\left\{T_{1},T_{2},\ldots ,T_{N_{t}}\right\}=\left\{T_{1}^{C},T_{2}^{C},\ldots ,T_{N_{C}}^{C},T_{N_{C+1}}^{D},T_{N_{C+2}}^{D},\ldots ,T_{N_{C}+N_{D}}^{D}\right\},$$

where $N_{C}$ and $N_{D}$ denote the numbers of type-C and type-D tasks, respectively, and $N_{t}$ is the total number of tasks.

Given $N_{t}$ tasks and $N_{u}$ UAVs, the objective is to optimize UAV–task assignments to maximize the global reward. The task allocation problem is mathematically defined in Equation (3).

(3) $$\max\sum_{i=1}^{N_{u}}\sum_{j=1}^{N_{t}}c_{ij}x_{ij},$$

subject to:

(4) $$\sum_{j=1}^{N_{t}}x_{ij}\leq L_{i}\;\forall i\in U,$$

(5) $$\sum_{i=1}^{N_{u}}x_{ij}\geq num_{j}\;\forall j\in T,$$

(6) $$\sum_{i=1}^{N_{u}}\sum_{j=1}^{N_{t}}x_{ij}\leq N_{\min}\in \left\{N_{t},N_{u}\times L_{i}\right\},$$

(7) $$TW_{j}=\left[t_{j}^{\mathrm{start}},t_{j}^{\mathrm{end}}\right],$$

where $N_{u}$ and $N_{t}$ represent the lengths of the UAV and task lists, respectively. $c_{ij}$ is the reward for UAV *i* executing task *j*, and $x_{ij}$ is the decision variable: $x_{ij}=1$ if task *j* is assigned to UAV *i*, otherwise $x_{ij}=0$. $L_{i}$ is the maximum number of tasks UAV *i* can handle, and $num_{j}$ is the minimum number of UAVs required for task *j*. $N_{\min}$ is the maximum number of tasks the UAV system can execute, and $TW_{j}$ defines the time window for task *j*.

Although the task-allocation problem can be mathematically formulated as a 0–1 programming model, the main difficulty of this research does not lie in solving a conventional binary optimization problem. Rather, it lies in efficiently handling discrete task-allocation decisions in highly dynamic, heterogeneous, and communication-constrained multi-UAV environments. Traditional 0–1 optimization and centralized approaches depend on complete global information and fixed network topologies, which limits their scalability and adaptability to large-scale, time-varying UAV networks. To overcome these limitations, this paper proposes the TLC-CBBA framework, which integrates hierarchical clustering with distributed consensus to jointly optimize the communication structure, resource balance, and task–resource matching, enabling scalable, real-time, and conflict-free task allocation.

### 3.2. Consensus-Based Bundle Algorithm

The CBBA operates in two alternating phases: task bundle construction and consensus. In the construction phase, each UAV incrementally selects tasks using a greedy strategy to maximize its individual reward. The consensus phase resolves conflicts via communication with neighboring UAVs.

The main components of the algorithm include:

(1) Bundle list $b_{i}$: tasks assigned to UAV *i*, recorded in the order of selection;
(2) Path list $p_{i}$: the sequence in which UAV *i* executes its tasks;
(3) Bid list $y_{i}$: the highest bid placed by UAV *i* for each task;
(4) Assignment list $z_{i}$: the UAV designated to execute each task;
(5) Timestamp $s_{i}$: the most recent communication time between UAV *i* and others.

During bundle construction, UAVs iteratively add tasks to maximize reward. In the consensus phase, they exchange information to achieve conflict-free allocations. After communicating with UAV *k*, UAV *i* updates its decisions according to Equation (8).

(8) $$\left\{\begin{array}{c} \mathrm{Update}:\;y_{ij}=y_{kj},\;z_{ij}=z_{kj}\\ \mathrm{Reset}:\;y_{ij}=0,\;z_{ij}=\emptyset \\ \mathrm{Leave}:\;y_{ij}=y_{ij},\;z_{ij}=z_{ij} \end{array}\right.$$

**Figure 1:**
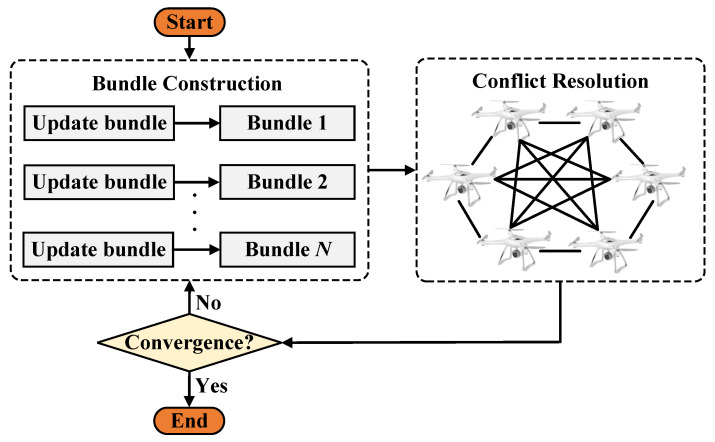
Flowchart of the CBBA.

UAVs use a lookup table to decide whether to update, reset, or leave their bid. When receiving a bid from UAV *k*, UAV *i* checks if it can win the task. If so, UAV *i* updates its bid and may take over tasks previously assigned to others. This process ensures that UAVs maintain updated situational awareness and resolve conflicts effectively. The CBBA flow is illustrated in Figure 1.

## 4. The Proposed Method

In this section, we provide a detailed description of the proposed TLC-CBBA, including the first-layer clustering strategy on communication network node grouping, the second-layer clustering strategy employing K-medoids with distance and resource balancing, and the overall implementation of TLC-CBBA.

### 4.1. First-Layer Clustering: A Communication Network-Based Node Grouping Strategy

In large-scale UAV cooperative systems, the traditional fully-connected communication architecture, while capable of ensuring complete information sharing, suffers from excessive bandwidth consumption and redundant data transmission. As the number of UAVs increases, the communication overhead grows at a rate of $O(N^{2})$, leading to communication bottlenecks, increased latency, and potential system instability. Therefore, constructing an efficient and scalable hierarchical communication architecture is essential for improving overall system performance.

Studies in network science have shown that nodes in a network exhibit structural heterogeneity: certain nodes occupy critical topological positions and possess higher centrality scores. By leveraging these structurally important nodes as communication backbones, it is possible to reduce communication overhead while maintaining global synchronization performance.

To this end, we propose a multi-metric centrality-based hierarchical clustering approach. UAVs are assigned different functional roles according to their structural importance within the communication graph, including global synchronization nodes (core nodes), local broadcasting nodes, and relay nodes. The overall procedure consists of the following stages:

(i) A small subset of UAVs with the highest centrality scores is selected as core nodes to form the communication backbone;
(ii) A sparse and robust global synchronization topology is constructed among the core nodes;
(iii) Each core node aggregates global information and broadcasts it to its associated local cluster members, enabling efficient propagation from core to edge.

Furthermore, all non-core UAVs are assigned to their corresponding core clusters based on the shortest path distances to each core node, resulting in a structured and efficient grouping scheme. This design reduces the overall communication complexity from $O(N^{2})$ to $O(K^{2}+N\cdot K)$, where *K* is the number of selected core nodes and K≪N, significantly improving the system’s scalability and responsiveness.

The structural importance of each UAV is quantified by a composite centrality score $C_{i}$, which is formulated as a weighted combination of four classical centrality metrics (Equation (9)).

(9) $$C_{i}=\sum_{k=1}^{4}w_{k}\cdot C_{i}^{\left(k\right)},$$

where $C_{i}^{\left(k\right)}$ represents the *k*-th centrality metric (degree, closeness, betweenness, and eigenvector centrality), and $w_{k}$ denotes the corresponding weight. Based on the parameter sensitivity analysis in Section 5.5, $w_{k}$ is set to 0.25, though it can be flexibly adjusted according to specific mission requirements.

Additionally, if UAV type information and resource vectors are available, the system performs a resource-aware adjustment during the grouping process. UAVs of different types are evenly distributed across core clusters based on both type balance and resource load to prevent redundancy and imbalance. This multi-stage clustering strategy not only improves communication efficiency but also accounts for system heterogeneity and functional diversity, thereby laying a solid foundation for the second-layer clustering and subsequent task allocation.

The complete pseudocode of this process is provided in Algorithm 1.

### 4.2. Second-Layer Clustering: A Resource-Balanced and Distance-Aware K-Medoids Clustering Strategy

While the first-layer clustering identifies central UAVs that act as communication hubs, practical multi-UAV systems are inherently heterogeneous—UAVs differ in sensing capabilities, payloads, energy reserves, and mission roles (e.g., surveillance, relay, strike). Ignoring this heterogeneity during task allocation or sub-clustering may lead to imbalanced resource usage, functional mismatches, and overloading of certain nodes. Furthermore, spatial distribution plays a crucial role in determining communication latency and task responsiveness. To this end, we introduce a second-layer clustering strategy that forms resource-efficient and spatially compact subgroups under each hub, thereby improving the cooperative performance of UAVs.

#### 4.2.1. K-Medoids Clustering Process

Clustering partitions samples into subsets based on similarity or dissimilarity. K-medoids clustering selects representative medoids as cluster centers and assigns other samples to their nearest medoid, thereby minimizing the overall clustering loss.

Let there be *n* samples $X=\{x_{1},x_{2},\ldots ,x_{n}\}$ to be divided into *k* clusters $C=\{c_{1},c_{2},\ldots ,c_{k}\}$. The objective function is expressed in Equation (10).

(10) $$J=\sum_{i=1}^{k}\sum_{x_{j}\in C_{i}}{∥x_{j}-\mu_{c_{i}}∥}_{p},$$

where $\mu_{c_{i}}$ is the medoid of cluster $C_{i}$, and *p* denotes the norm (set to p=2 in this work).

The K-medoids algorithm proceeds as follows:

Step 1: Randomly select *k* samples as the initial medoids;

Step 2: Assign each remaining sample to its nearest medoid;

Step 3: For each cluster, compute the total distance of each member to all other members and update the medoid to the sample with the minimal distance sum.

These steps are iteratively repeated until the medoids stabilize.
**Algorithm 1:** Network Centrality-based UAV Clustering.
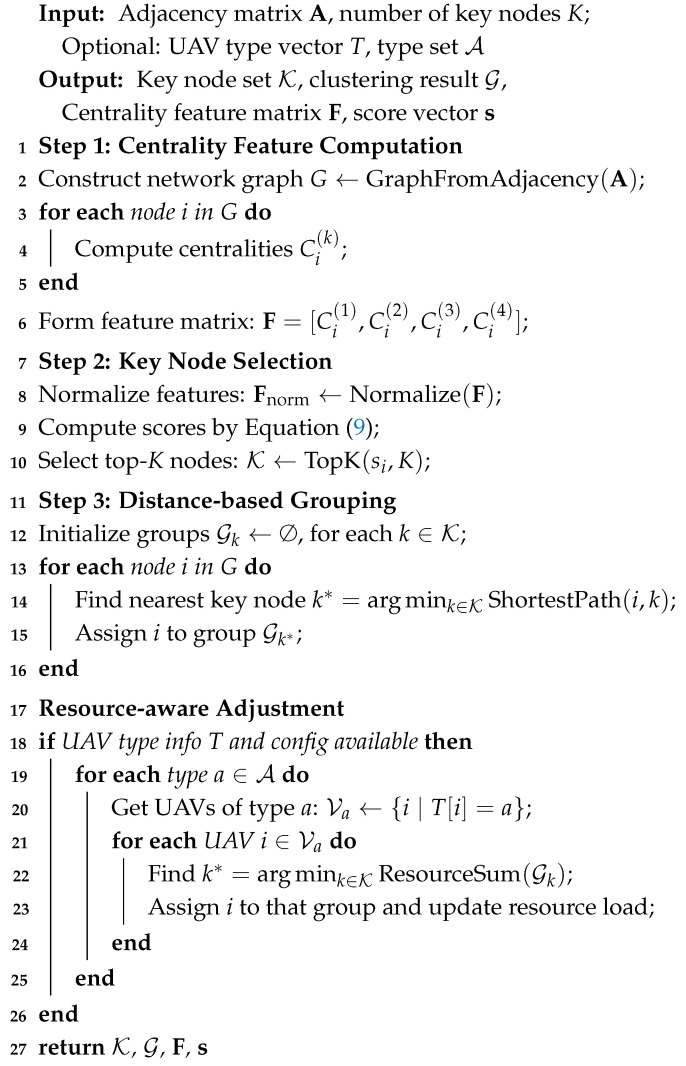


#### 4.2.2. UAV Clustering with Resource Balance

In addition to spatial compactness, maintaining functional balance across UAV clusters is crucial for ensuring fair and efficient task execution. Each UAV cluster $C_{i}$ is therefore associated with a set of task-relevant resources, as defined in Equation (11).

(11) $$r_{C_{i}}=\left\{r_{C_{i}}^{1},r_{C_{i}}^{2},\ldots ,r_{C_{i}}^{m}\right\},$$

where $r_{C_{i}}^{q}$ denotes the aggregated quantity of the *q*-th resource dimension available within cluster $C_{i}$.

The degree of balance is quantified by computing the standard deviation of the resource distribution within each cluster, as formulated in Equation (12).

(12) $$B_{r_{C_{i}}}=\sqrt{\frac{1}{m}\sum_{q=1}^{m}{\left(r_{C_{i}}^{q}-{\bar{r}}_{C_{i}}\right)}^{2}},$$

where the mean resource level is given by

(13) $${\bar{r}}_{C_{i}}=\frac{1}{m}\sum_{q=1}^{m}r_{C_{i}}^{q}.$$

*Example.* Consider a cluster consisting of three UAVs with available energy levels of [80,85,90] units. According to Equation (12), the calculated balance coefficient is $B_{r_{C_{i}}}=0.04$, indicating a highly uniform resource distribution. By contrast, another cluster with energy levels of [60,85,110] yields $B_{r_{C_{i}}}=0.23$, reflecting a greater degree of imbalance. Therefore, a smaller $B_{r_{C_{i}}}$ signifies a more balanced allocation of resources within the cluster, mitigating potential bottlenecks (e.g., clusters dominated by UAVs with high endurance but limited sensing capability).

Such a balance is essential in heterogeneous UAV systems, where mission requirements often demand complementary capabilities for cooperative execution. The clustering objective of the Resource-Balanced and Distance-Aware K-medoids Clustering Strategy, which jointly accounts for functional balance and spatial compactness, is formulated as presented in Equation (14).

(14) $$P_{2}=\gamma_{1}\sum_{i=1}^{k}B_{r_{C_{i}}}+\gamma_{2}{∥x_{i}-\mu_{c_{i}}∥}_{2},$$

where $\gamma_{1}$ and $\gamma_{2}$ are weighting coefficients that control the trade-off between resource uniformity and spatial compactness. The two terms in Equation (14) are on comparable scales because both are normalized before being combined. Specifically, the resource balance index $B_{rC_{i}}$ is a dimensionless quantity obtained through variance-based normalization of aggregated resource levels, while the spatial compactness term $∥x_{i}-\mu_{C_{i}}{∥}_{2}$ is normalized by the maximum communication range within each cluster. This normalization ensures that both terms contribute proportionally to the overall objective.

In this study, $\gamma_{1}$ and $\gamma_{2}$ are non-negative and satisfy the linear constraint $\gamma_{1}+\gamma_{2}=1$. By adjusting their ratio, TLC-CBBA can emphasize either resource balance (with a larger $\gamma_{1}$) or spatial compactness (with a larger $\gamma_{2}$), depending on specific mission priorities. Based on the parameter sensitivity analysis in Section 5.5, we set $\gamma_{1}=\gamma_{2}=0.5$ to assign equal importance to both aspects and achieve a balanced trade-off. This weighting scheme also enhances the consistency and comparability of experiments, since both factors are dimensionally consistent and properly normalized.

The implementation of this second-layer clustering strategy is outlined in Algorithm 2.

### 4.3. Implementation of the Two-Level Clustered Consensus-Based Bundle Algorithm

This section details the implementation of the proposed TLC-CBBA, which enables efficient task allocation in large-scale UAV networks by leveraging hierarchical clustering, distributed bundle construction, and consensus-based conflict resolution. The algorithm proceeds through five phases: (i) First-layer clustering, (ii) Second-layer clustering, (iii) Bundle construction, (iv) Conflict resolution, and (v) Objective optimization.
**Algorithm 2:** K-Medoids Clustering with Resource Balance and Spatial Compactness.
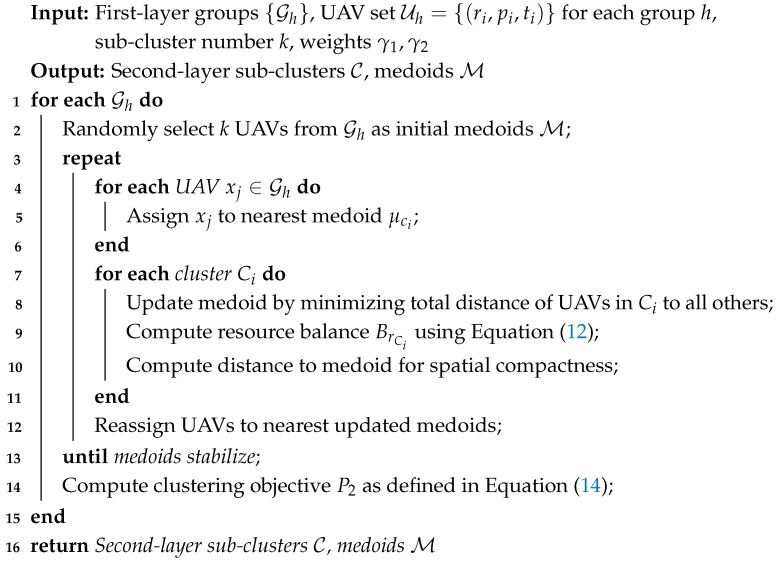


#### 4.3.1. First-Layer Clustering

The UAV network is organized based on network centrality into a three-tiered communication hierarchy—consisting of core, global synchronization, and local broadcast layers—as described in Section 4.1. UAVs with high centrality scores are selected as communication backbones to support efficient, scalable task coordination.

#### 4.3.2. Second-Layer Clustering

Each core node, obtained from the first-layer communication-based clustering, further partitions its associated UAVs into subgroups that are both resource-balanced and spatially compact. This second-layer clustering is performed using the K-medoids algorithm, where distances are computed as a weighted combination of resource and position features (Equation 10). To ensure fairness in heterogeneous UAV systems, type-aware balancing can optionally be applied so that UAV types are equitably distributed across sub-clusters. Formally, let $U=\{u_{1},\ldots ,u_{N}\}$ denote all UAVs under a given core node, and $C=\{C_{1},\ldots ,C_{K}\}$ represent the resulting sub-clusters, each led by a medoid node $u_{k}^{\mathrm{leader}}$. The overall clustering objective integrates both resource balance and spatial compactness, as defined in Equation (14).

#### 4.3.3. Bundle Construction Phase

In the TLC-CBBA, each UAV independently constructs its task bundle using a greedy strategy that iteratively selects tasks maximizing a utility score. The bundle construction process involves two key steps: (i) removing tasks for which the UAV has been outbid, and (ii) appending new tasks that offer the highest score.

(1) Basic reward function with time decay

To evaluate the time sensitivity of each task, we define a reward function that incorporates temporal decay. This encourages UAVs to prioritize high-value tasks that can be completed earlier, as shown in Equation (15).

(15) $${\mathrm{score}}_{ij}=q_{j}\cdot \exp\left(-\lambda_{j}\cdot \max\left(0,s_{ij}^{\min}-a_{j}\right)\right),$$

where $q_{j}$ is the intrinsic value of task *j*, $\lambda_{j}$ is the temporal decay coefficient, $a_{j}$ is the earliest start time of task *j*, and $s_{ij}^{\min}$ is the earliest feasible start time for UAV *i* to perform task *j*.

(2) Earliest start time calculation

The earliest start time $s_{ij}^{\min}$ is determined based on UAV *i*’s availability and travel time. If there is no predecessor task, as shown in Equation (16).

(16) $$s_{ij}^{\min}=\max\left\{a_{j},{\mathrm{availability}}_{i}+\frac{∥p_{i}-l_{j}∥}{v_{i}}\right\},$$

where $p_{i}$ is the current position of UAV *i*, $l_{j}$ is the task location, and $v_{i}$ is the UAV’s speed.

If task *j* is associated with a predecessor task *k* in the current bundle, the relationship is formulated in Equation (17).

(17) $$s_{ij}^{\min}=\max\left\{a_{j},s_{ik}+d_{k}+\frac{∥l_{k}-l_{j}∥}{v_{i}}\right\},$$

where $d_{k}$ is the duration of task *k*.

(3) Load balancing penalty

To avoid overloading high-capacity UAVs, a nonlinear load-penalty term is introduced in the objective. When the number of tasks assigned to a UAV reaches or exceeds its maximum capacity, the penalty is set to zero; otherwise, the penalty decays exponentially with the number of assigned tasks. The penalty intensity is controlled by a tunable parameter α. This mechanism discourages over-reliance on a small subset of UAVs and promotes a more balanced task distribution across the fleet.

(4) Compatibility constraint

To ensure that each UAV can feasibly perform a given task in terms of type and capability, a binary compatibility constraint is defined. This constraint relies on a predefined compatibility matrix. If the compatibility value between a UAV’s type and a task’s type exceeds a threshold (e.g., 0.5), the UAV is considered capable of executing the task; otherwise, the task is deemed infeasible for that UAV. This prevents task assignment failures caused by resource or functionality mismatches.

(5) Time window feasibility check

Beyond type and load considerations, tasks must also comply with their time window requirements. Specifically, the earliest feasible start time for a UAV to execute a task must fall within the allowable time interval. This interval is determined by the task’s earliest start time and its latest finish time minus its duration. This feasibility check ensures that all assigned tasks can be completed not only in terms of resources but also within the temporal constraints.

(6) Final task score

By integrating the temporal reward, load balancing penalty, and compatibility constraints, the task score is formally defined in Equation (18).

(18) $${\mathrm{score}}_{ij}=q_{j}\cdot \exp\left(-\lambda_{j}\cdot \max\left(0,s_{ij}^{\min}-a_{j}\right)\right)\cdot \exp(-\alpha \cdot |B_{i}\left|\right)\cdot I\left[{\mathrm{compatibility}}_{ij}>0.5\right],$$

where $B_{i}$ denotes the bundle set of UAV *i*, containing the tasks currently assigned to it, and $|B_{i}|$ represents the number of these tasks. The exponential term $\exp(-\alpha |B_{i}|)$ penalizes excessive task accumulation on a single UAV, encouraging a more balanced workload across the swarm. The hyper-parameter α controls the penalty strength—smaller values yield weaker load regulation and higher utilization, whereas larger values enforce stricter balancing. In this paper, α=0.3 is empirically set to ensure stable performance and a reasonable trade-off between efficiency and load balance. The temporal decay coefficient $\lambda_{j}$ controls the time-sensitivity of task *j*; larger $\lambda_{j}$ values impose stronger penalties on delayed execution. According to task priority, $\lambda_{j}$ is empirically set within [0.05, 0.5].

This multiplicative design ensures a task receives non-zero utility only when all feasibility conditions are met, thus improving stability and task selection robustness.

(7) Task selection strategy

From the set of candidate tasks, each UAV selects the one with the highest score. If multiple tasks share the same score, a priority rule applies: UAVs with smaller ID numbers are given precedence. If a tie still remains, the task with the earliest feasible start time is chosen. This mechanism ensures that when UAVs or tasks are otherwise similar, the system favors faster execution, thereby improving overall efficiency and responsiveness.

#### 4.3.4. Conflict Resolution

In the TLC-CBBA, the first-layer clustering is primarily designed for communication topology optimization. By constructing distributed synchronization pathways through key nodes, it reduces communication redundancy and improves transmission efficiency. This hierarchical structure provides an organizational foundation for second-layer K-medoids clustering, which focuses on resource-aware grouping.

The conflict resolution process is executed at the second-layer clustering layer and follows a three-stage mechanism to achieve consistent and scalable task allocation across the UAV network.

(1) Intra-cluster resolution

Within each K-medoids cluster, UAVs form a fully connected subnetwork and execute the standard CBBA (CBBA). Each UAV greedily constructs a task bundle based on local utility scores and exchanges bid information with neighbors.

If multiple UAVs bid for the same task, the task is awarded to the one with the highest bid. In case of a tie, a predefined priority rule (e.g., node ID) is applied to determine the winner, ensuring rapid convergence within the cluster.

(2) Inter-cluster coordination

To prevent task duplication across clusters, medoid nodes of each cluster communicate with one another to synchronize bid results. If a task is selected in multiple clusters, the system compares bids and assigns the task to the UAV with the highest global bid. Other clusters then remove the task from their candidate list and update their local bundles accordingly.

(3) Inter-group synchronization

Key nodes of each communication group are responsible for propagating the final assignment results across the entire network. This synchronization is conducted through the backbone formed by the first-layer clustering:

(i) Intra-group aggregation: Key nodes collect final task decisions from all clusters within the group;

(ii) Inter-group exchange: Key nodes communicate globally to resolve cross-group inconsistencies;

(iii) Hierarchical broadcast: Finalized assignments are disseminated to all UAVs via medoid and key node relays.

### 4.4. Objective Function

The objective of TLC-CBBA is to maximize the overall utility of the UAV system while satisfying a set of feasibility constraints. The global optimization problem is formulated as follows, as shown in Equation (19).

(19) $$\max_{B}\;\sum_{i=1}^{N}\sum_{j\in B_{i}}{\mathrm{score}}_{ij},$$

where the ${\mathrm{score}}_{ij}$ is defined in Equation (18). The optimization is subject to the following constraints:

(20) $$\begin{array}{cc} & \sum_{i=1}^{N}x_{ij}\leq 1,\;\forall j\in T, \end{array}$$

(21) $$\begin{array}{cc} & |B_{i}|\leq m_{i}^{\max},\;\forall i\in U, \end{array}$$

(22) $$\begin{array}{cc} & a_{j}\leq s_{ij}^{\min}\leq b_{j}-d_{j},\;\forall i,j:x_{ij}=1, \end{array}$$

(23) $$\begin{array}{cc} & s_{ij}^{\min}\geq s_{ik}+d_{k}+\frac{∥l_{k}-l_{j}∥}{v_{i}},\;\forall i,k,j:x_{ik}=x_{ij}=1, \end{array}$$

(24) $$\begin{array}{cc} & x_{ij}=0,\;\mathrm{if}\;\mathrm{compatibility\_mat}\left[t_{i}\right]\left[t_{j}\right]\leq 0.5, \end{array}$$

where U and T denote the sets of UAVs and tasks with cardinalities |U|=N and |T|=M, respectively; $x_{ij}$ is the binary decision variable indicating whether UAV *i* is assigned to task *j*; $q_{j}$ is the base value of task *j*; $s_{ij}^{\min}$ is the earliest feasible start time of UAV *i* for task *j*; $a_{j}$ and $b_{j}$ denote the lower and upper bounds of the time window of task *j*; $d_{j}$ is the task duration; $l_{j}$ is the spatial location of task *j*; $v_{i}$ is the flight speed of UAV *i*; α is the penalty factor for load balancing; $m_{i}^{\max}$ is the maximum task capacity of UAV *i*; and $\mathrm{compatibility\_mat}\left[t_{i}\right]\left[t_{j}\right]$ encodes the compatibility between UAV type $t_{i}$ and task type $t_{j}$.

**Figure 2:**
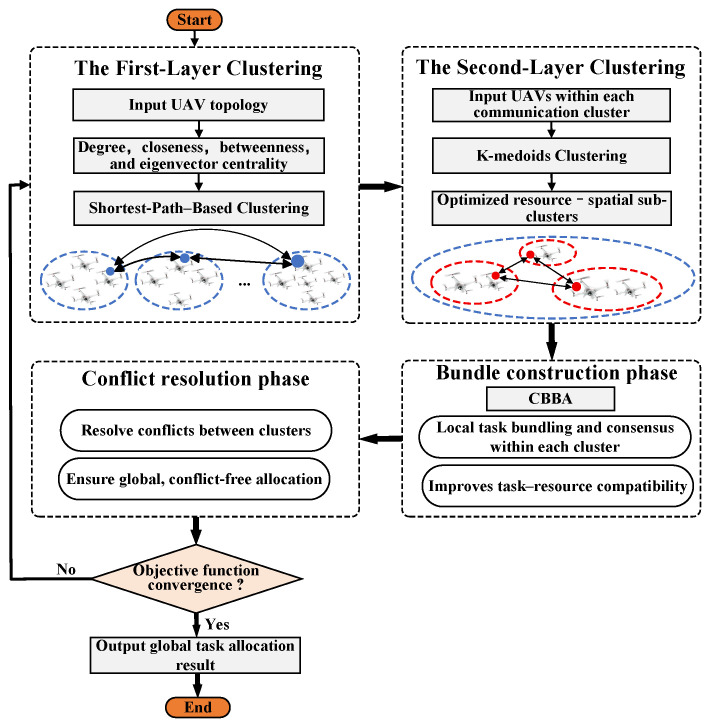
Flowchart of the proposed TLC-CBBA.

TLC-CBBA unifies clustering, bundle construction, and conflict resolution into a single optimization framework. By leveraging hierarchical communication and distributed consensus, it achieves scalable and efficient task allocation across large UAV teams. The complete procedure is illustrated in Algorithm 3 and Figure 2.
**Algorithm 3:** Two-Level Clustered CBBA (TLC-CBBA).
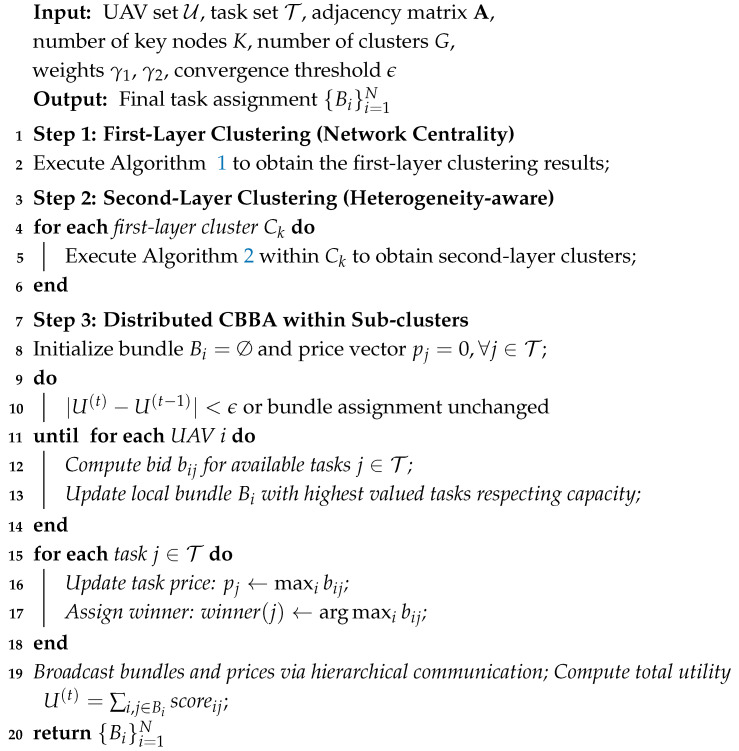


### 4.5. Analysis of Dynamic Adaptability

The proposed TLC-CBBA is inherently designed with adaptive mechanisms to handle dynamic mission environments. When task distributions, UAV states, or communication topologies undergo substantial changes, the system triggers on-demand re-clustering to maintain balanced cluster structures and stable task assignments.

Re-clustering may be initiated in the following situations: (i) when a subset of UAVs loses communication with its cluster head; (ii) when newly generated tasks significantly increase the workload, disturbing the existing balance; or (iii) when inter-cluster communication delays rise significantly, degrading synchronization efficiency. During these events, the system monitors several indicators—re-clustering latency, control-message overhead, and the proportion of reassigned UAVs or tasks—to assess its responsiveness, coordination cost, and update scope.

By confining re-clustering operations to locally affected clusters, TLC-CBBA avoids global recomputation, thereby preserving overall coherence and communication efficiency under mission or network disturbances. The framework’s hierarchical clustering and localized consensus mechanisms further enable it to flexibly adapt to dynamic conditions such as task arrivals, UAV failures, and temporary communication interruptions. These design features collectively demonstrate the strong robustness, responsiveness, and scalability of TLC-CBBA, establishing a solid methodological foundation for real-world cooperative UAV applications.

## 5. Simulation and Analysis

In this section, we present the simulation setup for multi-UAV task allocation, covering task and UAV configuration, network topology, and parameter settings. We then present a validation of the effectiveness of the first-layer clustering in TLC-CBBA to demonstrate its rationality, followed by an analysis of the second-layer clustering results, an overall performance evaluation of TLC-CBBA, and a significance analysis comparing TLC-CBBA with other benchmark algorithms.

### 5.1. Simulation Setup

#### 5.1.1. Task and UAV Configuration

The simulation environment consists of 25 UAVs, including 9 attack UAVs, 8 transport UAVs, and 8 reconnaissance UAVs, each equipped with heterogeneous resource attributes such as payload, fuel, and capacity. UAVs are initialized at fixed positions on a predefined two-dimensional grid, with a maximum task capacity of 10 and an initial fuel level of 1000. A total of 30 tasks are generated, evenly distributed across the three categories (attack, transport, reconnaissance) to align with UAV types. Tasks are uniformly placed within a three-dimensional space of 400 × 400 × 400. Each task is associated with an independent time window, randomly sampled from the global horizon [0, 1000], with widths ranging from 50 to 200 time units. These settings are kept consistent throughout the study, and any adjustments made for specific experiments are clearly indicated where applicable. The TLC-CBBA is evaluated under various network topologies, including fully connected, star, tree, ring, and sparse random structures.

#### 5.1.2. Comparison Algorithm and Parameter Settings

The performance of TLC-CBBA is assessed through comprehensive simulations against DMCHBA [27], G-CBBA [44], standard CBBA [36], and Clustering-CBBA [8]. Each setup is repeated 50 times under Monte Carlo trials to ensure statistical robustness. Baseline algorithm parameters are configured according to their original literature, whereas TLC-CBBA’s key settings are listed beneath the relevant equations and further examined in Section 5.5. All simulations are executed on a Microsoft Windows 11 Pro (64-bit) workstation with an Intel Core i5-10600 CPU and eight NVIDIA RTX 3060 GPUs, using Python 3.9.10 and PyTorch 1.8.1. The implementation of TLC-CBBA is openly accessible at: https://github.com/ycchao0406/TLC_CBBA (accessed on 29 October 2025).

### 5.2. Validation of the Effectiveness of the First-Layer Clustering in TLC-CBBA

This subsection verifies the rationality of the TLC-CBBA. Three types of UAVs and six representative communication network topologies are selected. For each network topology, 50 Monte Carlo simulations are conducted. By comparing the communication scale of the CBBA and its variants, the necessity of selecting key nodes in TLC-CBBA is analyzed, as well as the impact of different numbers of key nodes on the communication scale.

**Figure 3:**
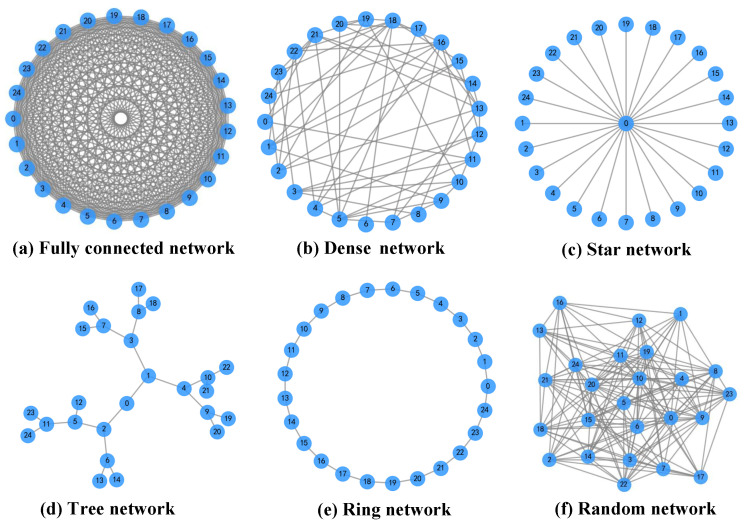
Six representative communication topologies: (**a**) Fully connected network, where every node is connected to all other nodes; (**b**) Dense network, in which each node links to most of the others; (**c**) Star network, consisting of one central node connected to all peripheral nodes; (**d**) Tree network, organized in a hierarchical branching structure without loops; (**e**) Ring network, where each node connects to two neighboring nodes forming a closed loop; and (**f**) Random network, featuring irregular and randomly distributed connections among nodes.

In the experiment, the target locations are fixed to ensure that the TLC-CBBA achieves the same optimal allocation scheme as the original CBBA. Moreover, when the number of key nodes is set to one, the selected node must handle a large volume of information throughput and is highly vulnerable to targeted attacks, which makes the resulting network fragile; therefore, this case is not considered. The six representative network topologies are illustrated in Figure 3, and the corresponding key node selections are summarized in Table 1. The results indicate that, across different communication topologies, the same core key nodes are consistently selected in most cases. This ensures that TLC-CBBA is able to generate identical allocation schemes under varying conditions. These findings demonstrate the strong robustness of the algorithm and show that its key-node selection mechanism effectively captures the structural characteristics of the network.

**Figure 4:**
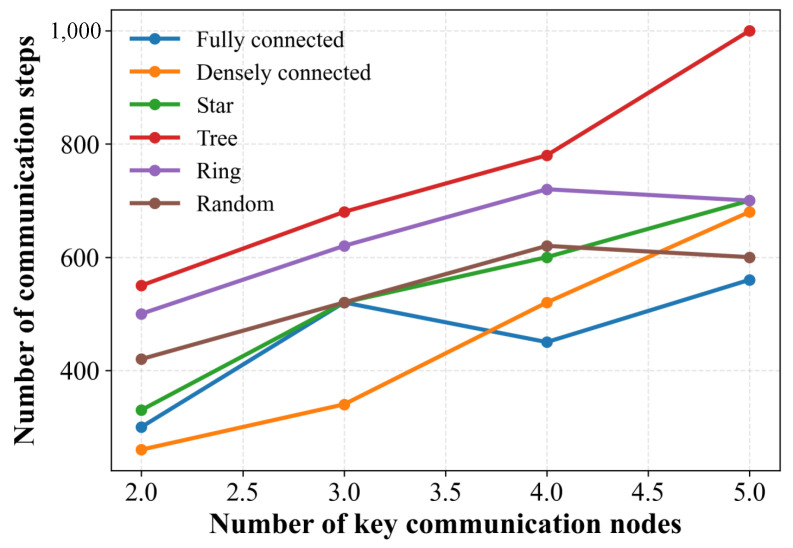
Effect of key node count on the communication frequency of TLC-CBBA across different topologies.

We further examine the communication frequency of different network topologies under varying numbers of key nodes. Figure 4 illustrates how the number of key nodes influences the communication frequency of TLC-CBBA across multiple topologies. The results indicate that communication overhead increases as the number of key nodes grows. This is because, in the first-layer clustering stage, each key node represents a group, and a larger number of groups inevitably requires more inter-group communication.

**Figure 5:**
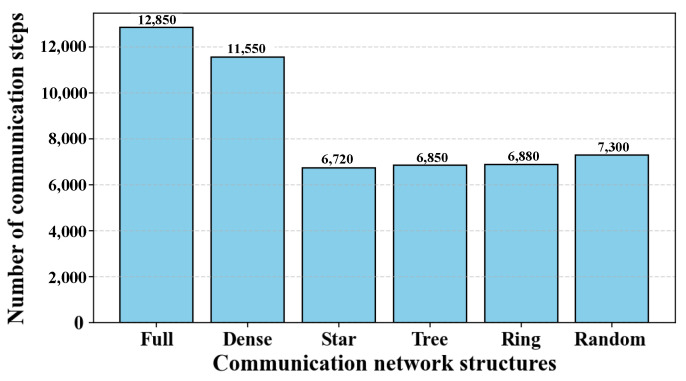
Baseline CBBA communication frequency under different topologies.

Figure 5 compares the number of communication steps of the baseline CBBA across different network topologies with the number of key nodes fixed at three. By combining the results in Figure 4 and Figure 5, it can be observed that TLC-CBBA consistently achieves significantly lower communication overhead than CBBA under the same conditions. This advantage arises from its hierarchical grouping mechanism, where inter-group communication is restricted to key nodes, thereby significantly reducing the overall communication cost.

### 5.3. Validation of the Effectiveness of the Second-Layer Clustering in TLC-CBBA

**Figure 6:**
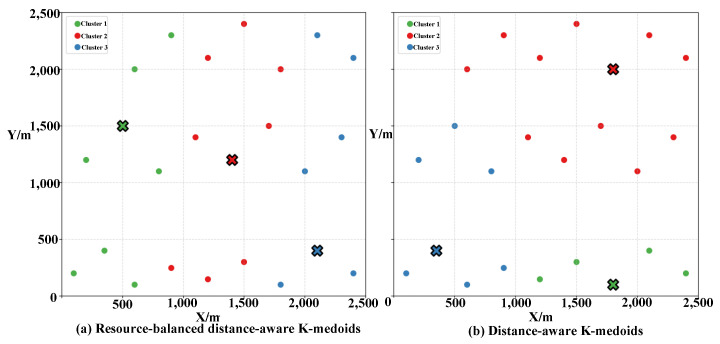
Clustering results of two K-medoids algorithms: (**a**) Resource-balanced distance-aware K-medoids algorithm; (**b**) Distance-aware K-medoids algorithm. The crosses (×) represent the cluster centroids (medoids) of each group.

To validate the effectiveness of the proposed second-layer resource-balanced distance-aware K-medoids clustering strategy, a second-layer clustering is performed within each communication group obtained from the first-layer clustering. The resulting cluster configurations are illustrated in Figure 6a, and the detailed UAV assignments and corresponding resource distributions are presented in Table 2. Compared with the distance-only K-medoids (Table 3), the proposed method keeps UAVs within each cluster spatially compact while achieving a more uniform resource distribution across clusters. Moreover, the total amount of weapon resources remains relatively consistent among clusters, which improves task-allocation efficiency in the consensus phase and enhances the overall rationality and effectiveness of the allocation scheme.

To further demonstrate the improvement in inter-cluster resource balance, a quantitative comparison between the distance-only K-medoids and the resource-balanced K-medoids is conducted under the same experimental conditions. As shown in Figure 6 and Table 2 and Table 3, the distance-only approach produces an imbalanced clustering outcome—particularly, the second cluster contains substantially more UAVs and weapon resources than the others—whereas the proposed resource-balanced version achieves a noticeably more even distribution.

According to Equation (12), the balance coefficient $B_{r(C_{i})}$ is computed as the standard deviation of the aggregated resource levels (payload, fuel, and capacity) within each cluster. Since the absolute magnitudes of $B_{r(C_{i})}$ depend on the resource units, the coefficients are further normalized to enable meaningful comparison across different clustering strategies. After normalization (where 0 denotes perfect balance and 1 denotes the worst imbalance), the average normalized balance coefficient decreases from approximately 0.21 (for the distance-only K-medoids) to about 0.09 (for the resource-balanced K-medoids), corresponding to an improvement of roughly 55%. This result confirms that incorporating resource-awareness into the K-medoids objective effectively enhances inter-cluster resource uniformity while maintaining spatial compactness. This result confirms that incorporating resource awareness into the K-medoids objective effectively enhances inter-cluster resource uniformity while preserving spatial compactness. The remaining minor differences among clusters stem from the intrinsic heterogeneity of UAV capabilities and the inherent trade-off between spatial compactness and resource balance imposed by the clustering objective. Therefore, perfect equality across clusters is neither realistic nor necessary in practical multi-UAV systems.

### 5.4. Overall Performance Analysis of TLC-CBBA

This subsection compares the TLC-CBBA algorithm with DMCHBA [27], G-CBBA [42], the standard CBBA [36], and Clustering-CBBA [8] in terms of task reward and communication scale. The evaluation is first carried out under communication networks with varying densities, where the density is defined as the ratio of nonzero elements to the total number of elements in the adjacency matrix, as shown in Equation (25).

(25) $$\frac{\sum_{i=1}^{m}\sum_{j=1}^{m}a_{ij}}{m^{2}},$$

where *m* denotes the number of UAV nodes.

**Figure 7:**
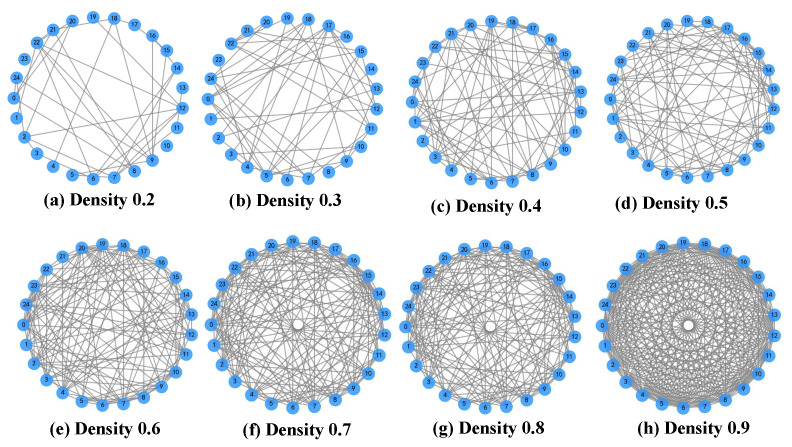
Communication topology graphs with different densities. Each blue circle represents a UAV node labeled by its ID number, and the network density reflects the sparsity level of connections among the nodes.

In the experiment, eight communication network structures with different densities are selected. For each structure, 50 topology graphs are randomly generated, with several examples shown in Figure 7.

**Figure 8:**
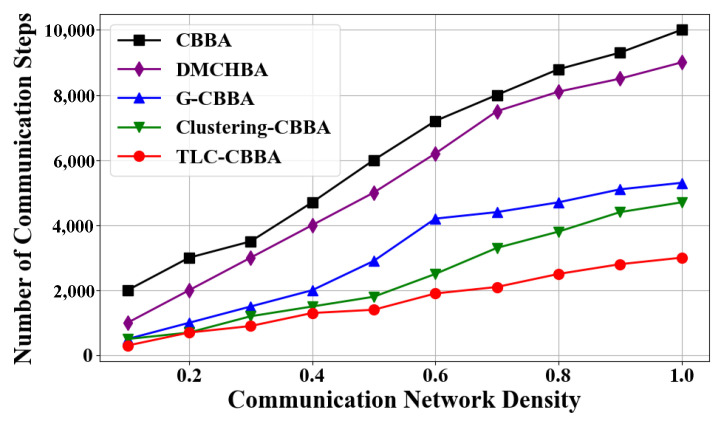
Communicationfrequency trends of different algorithms across network densities.

**Figure 9:**
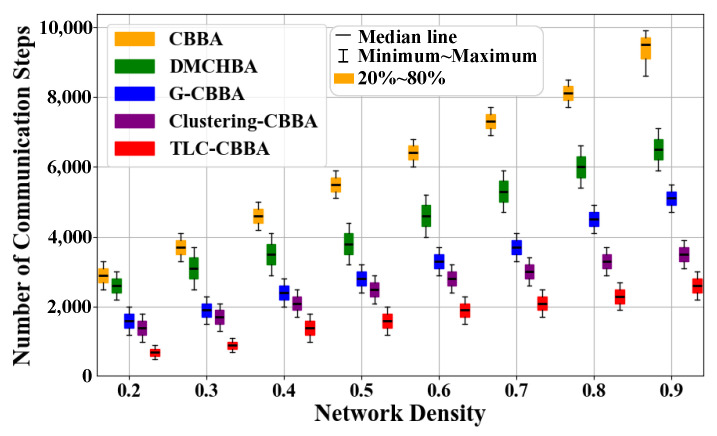
Comparisonof TLC-CBBA and four algorithms using box plots across different network densities.

Figure 8 presents the average total communication steps of TLC-CBBA compared with the other four algorithms under varying network communication densities. The results indicate that TLC-CBBA has significantly fewer communication steps than the other algorithms. Figure 9 shows the box plots of TLC-CBBA and the other four algorithms under different network communication densities. The results demonstrate that TLC-CBBA outperforms all other algorithms in terms of median, maximum, and minimum communication steps, with a smaller interquartile range (box size).

**Figure 10:**
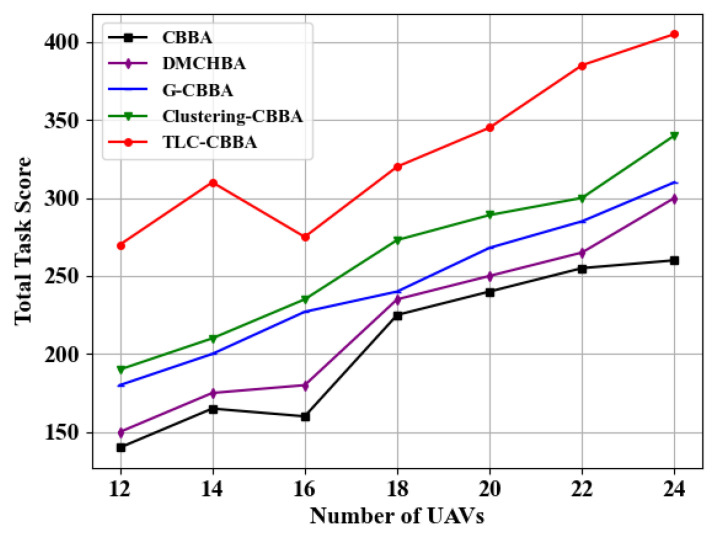
Comparisonof the total scores of different algorithms under varying numbers of UAVs.

In addition, with the number of tasks fixed at 30, a comparative evaluation is conducted in a fully connected network topology to assess the total score performance of TLC-CBBA across varying numbers of UAVs, and to compare it with four other algorithms. As shown in Figure 10, the experimental results indicate that TLC-CBBA consistently achieves higher total scores than the other four algorithms across different UAV scales, outperforming the latest algorithm, Clustering-CBBA, by 17.01% to 47.62%. Furthermore, Table 4 provides a detailed statistical evaluation and performance comparison of total task scores with 24 UAVs, where the bolded values indicate the optimal results. The results demonstrate that TLC-CBBA outperforms the other four algorithms in terms of average score, best score, worst score, CPU runtime (single run), and confidence interval.

**Figure 11:**
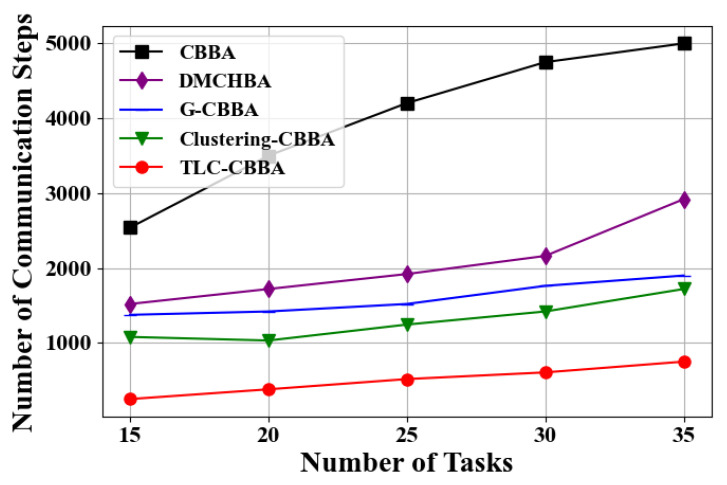
Comparison of the number of communication steps of different algorithms under varying numbers of tasks.

**Figure 12:**
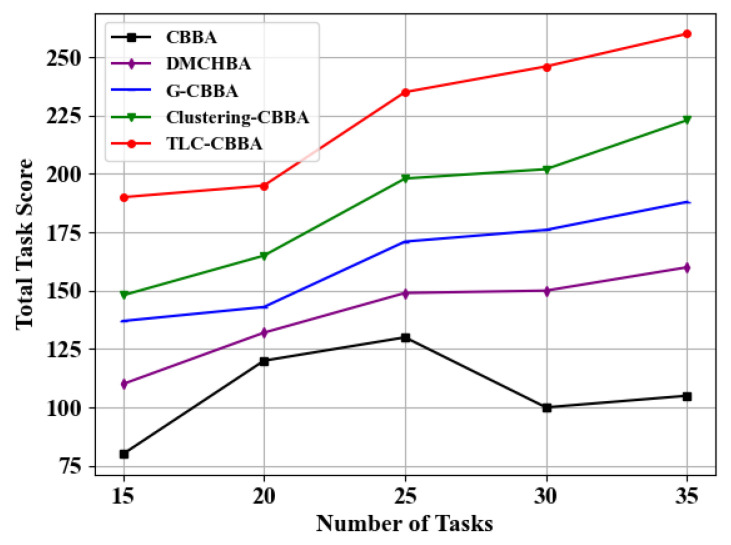
Comparison of the total task scores of different algorithms under varying numbers of tasks.

Similarly, we select 12 UAVs (4 transport UAVs, 4 reconnaissance UAVs, and 4 attack UAVs) in a fully connected network topology to evaluate the performance of TLC-CBBA against four other algorithms under varying task numbers (i.e., 15, 20, 25, 30, and 35). The results, shown in Figure 11 and Figure 12, demonstrate that TLC-CBBA consistently outperforms the other methods. Table 5 further presents the statistical comparison of communication steps across different algorithms on 25 tasks, including the average, best, and worst communication counts, CPU time (single run), and confidence intervals, where the bolded values indicate the optimal results. The results indicate that TLC-CBBA outperforms the other four algorithms in all these aspects. Besides, as illustrated in Figure 11, TLC-CBBA reduces the number of communication steps by 56% to 77% compared with the most recent method, Clustering-CBBA, and requires significantly fewer communication steps than the other three algorithms across different task numbers. As shown in Figure 12, TLC-CBBA improves the total task score by 18% to 28% compared with Clustering-CBBA, while also achieving higher scores than the other three algorithms under varying task numbers.

**Figure 13:**
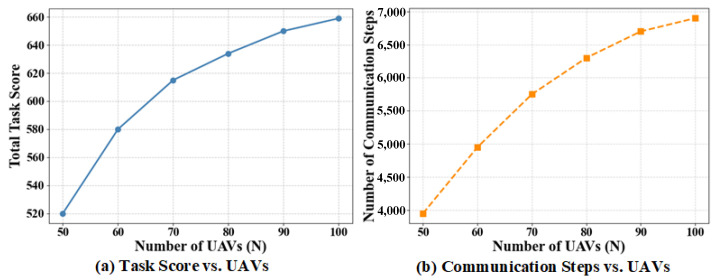
Variations of total task reward and communication cost with different numbers of UAVs: (**a**) Total task score vs. Number of UAVs; (**b**) Number of communication steps vs. Number of UAVs.

To verify the scalability of the proposed TLC-CBBA in large-scale UAV networks, the number of key nodes is fixed at 5 and a fully connected topology is adopted. The experiments are conducted under different swarm sizes (N=50,60,70,80,90,100) with the number of tasks fixed at 120. Figure 13 shows the variations of the total task reward and communication cost with respect to the number of UAVs. As the network scale increases, TLC-CBBA maintains stable overall performance; the total task reward increases gradually and controllably, while the number of communication rounds grows only slightly. These observations indicate that the proposed two-level clustering mechanism of TLC-CBBA effectively enhances communication hierarchy and task decomposition in large-scale UAV scenarios. The slowdown in the growth of the total task reward results from reduced task competition and the saturation of resource allocation as the swarm size increases.

### 5.5. Parameter Sensitivity Analysis

With the number of tasks fixed at 30 and the number of UAVs fixed at 15, a parameter sensitivity analysis is performed under a fully connected network topology to evaluate the performance of the TLC–CBBA under different weighting configurations. Specifically, the effects of varying the task–weight coefficient $w_{k}$ (0.1–0.5) and the trade-off parameters $\gamma_{1}$ and $\gamma_{2}$ (0.1–0.9) are examined in terms of the average total score, communication steps, and runtime.

Table 6 illustrates how $w_{k}$, $\gamma_{1}$, and $\gamma_{2}$ influence the average total score, average communication steps, and average runtime, respectively. The results indicate that when $w_{k}=0.25$, $\gamma_{1}=0.5$, and $\gamma_{2}=0.5$, the algorithm achieves the highest average total score, the fewest communication steps, and the shortest runtime, demonstrating a well-balanced trade-off between performance and efficiency.

### 5.6. Significance Analysis of TLC-CBBA Compared with Other Algorithms

To assess performance differences objectively, the Wilcoxon rank-sum test is used to evaluate runtime differences between TLC-CBBA and the other four algorithms across 30 independent runs. The experiment involves 12 UAVs (4 transport, 4 reconnaissance, and 4 attack) and 25 tasks in a fully connected network topology. Results, summarized in Table 7, are evaluated at a significance level of 0.05. A *p*-value below this threshold indicates statistical significance, while a *p*-value above 0.05 suggests no significant difference. Effect size further quantifies the magnitude of the difference: 0<effectsize≤0.2 indicates a small effect, 0.2<effectsize≤0.5 a medium effect, and 0.5<effectsize≤0.8 a large effect. The symbols “+”, “≈”, and “–” represent significant difference, no difference, and non-significance, respectively.

The outcomes show that TLC-CBBA consistently exhibits statistically significant improvements over all four algorithms (p<0.001). In particular, the effect sizes against CBBA, DMCHBA, and G-CBBA range from 0.82 to 0.86, which represent very large effects, while the effect size against Clustering-CBBA is 0.612, also corresponding to a large effect. These findings confirm that TLC-CBBA provides a substantial and consistent runtime advantage compared with the other algorithms.

In summary, the experimental results demonstrate that the proposed TLC-CBBA significantly outperforms the comparison algorithms in terms of communication efficiency, total task score, runtime, and significance analysis. Moreover, it maintains stable and efficient performance across different communication topologies and task scales, highlighting its strong generality and robustness.

### 5.7. Evaluation of Dynamic Adaptability Under Dynamic Environments

To evaluate the online responsiveness and adaptability of TLC–CBBA, a dynamic simulation scenario based on a fully connected topology is established. The system is initialized with 15 UAVs and approximately 30 tasks, and it runs for 50 discrete time steps, each representing an update of the system state, including task arrivals, UAV failures, and link variations. Task arrivals follow a Poisson distribution (λ=2.0) [54,55]; each UAV fails independently with a probability q=0.02 according to a Bernoulli process [56,57], and communication links drop randomly with a probability p=0.1 (random seed 42) [58]. Whenever any triggering condition is satisfied, the system performs the two level re-clustering and CBBA reallocation process, which includes: (i) periodic triggering every k=5 steps; (ii) task-driven triggering, activated when the number of newly added tasks is greater than or equal to τ=1; and (iii) event-driven triggering, activated when the UAV dropout or link-change ratio is greater than or equal to ρ=0.1.

The parameter settings are selected with reference to the cited literature and preliminary experiments to represent a typical medium-dynamics environment. Specifically, λ=2.0 corresponds to a moderate-load condition; q=0.02 and p=0.1 indicate an average node reliability of 98% and a medium level of communication disturbance; k=5 balances responsiveness and communication overhead, whereas τ=1 and ρ=0.1 ensure adequate sensitivity to topological variations. Empirical evaluation confirms that variations within these parameter ranges do not affect the overall performance trend of TLC-CBBA.

**Figure 14:**
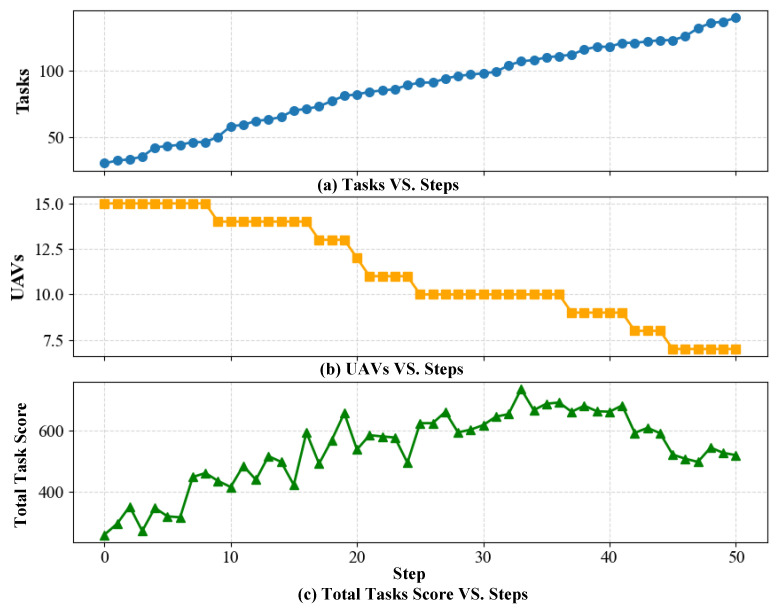
Variations of system states and performance under dynamic conditions: (**a**) Number of tasks vs. simulation steps; (**b**) Number of active UAVs vs. simulation steps; (**c**) Total task score vs. simulation steps.

As shown in Figure 14, the number of tasks increases monotonically with simulation steps, while the number of UAVs decreases stepwise due to random dropouts. Despite the gradual degradation of resources and connectivity, the proposed TLC–CBBA maintains sub-second responsiveness through event-triggered two-level re-clustering and reallocation. Consequently, the total task reward rises rapidly during the early phase and remains at a high level with slight fluctuations in the later phase (peaking at approximately $6.5\times 10^{2}$–$7.1\times 10^{2}$ and stabilizing around $5.5\times 10^{2}$–$6.5\times 10^{2}$). These results demonstrate that, under dynamic perturbations caused by Poisson task arrivals, UAV dropouts, and link failures, the proposed method exhibits strong responsiveness and stability, achieving smooth performance degradation rather than instability or collapse.

**Figure 15:**
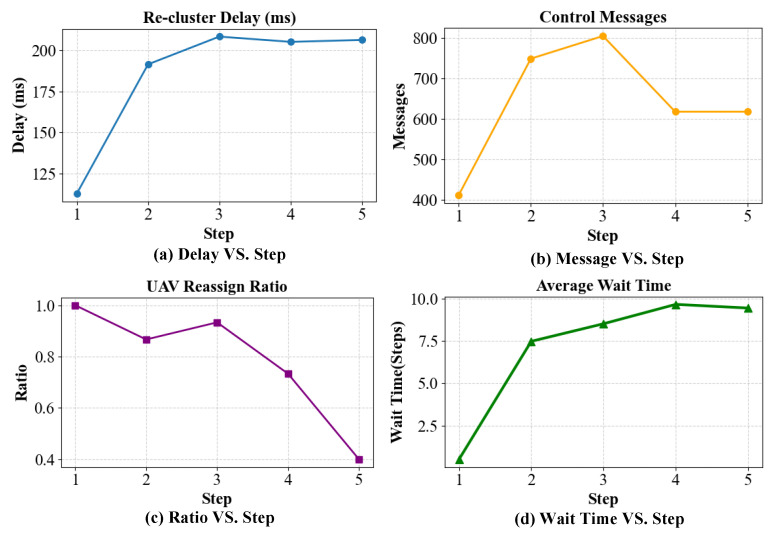
Variations of dynamic performance indicators during the first five re-clustering events: (**a**) Re-clustering delay vs. step; (**b**) Number of control messages vs. step; (**c**) UAV re-assignment ratio vs. step; (**d**) Average waiting time vs. step.

To further demonstrate the adaptability of the proposed TLC–CBBA under dynamic environments, a short-term online simulation is conducted that includes the first five re-clustering events, where disturbances are introduced by task arrivals and link variations. As shown in Figure 15, the re-clustering delay gradually increases from approximately 110 ms to 210 ms, indicating that the system requires slightly more time for reconfiguration as the network load and communication overhead grow. A similar trend is observed in the number of control messages, which peaks at around 800 during the third step and then stabilizes. The UAV re-assignment ratio remains above 0.8 for the first three steps, demonstrating rapid responsiveness to network changes, and then slightly decreases as the system reaches equilibrium. Meanwhile, the average waiting time increases from less than one step to approximately nine steps, reflecting the growth of scheduling delay caused by task accumulation. Although only the first five events are reported here, these quantitative variations clearly demonstrate the algorithm’s real-time adaptability and the controllability of its communication and computational overhead under dynamic disturbances.

Table 8 summarizes the results of the complete simulation under Poisson task arrivals (λ=2.0), UAV dropouts (q=0.02), and link failures (p=0.1). Over 50 time steps, the system triggers 49 re-clustering events. The average re-clustering delay is approximately 545 ms (sub-second level), while each event involves an average of about 346 control messages, corresponding to a communication volume of approximately $2.8\times 10^{5}$ bytes per event. The UAV and task re-assignment ratios are approximately 0.855 and 0.374, respectively, and the average waiting time is around 15 s. Overall, the proposed TLC–CBBA achieves a well-balanced trade-off among rapid responsiveness, low communication overhead, and moderate task re-assignment, effectively maintaining responsiveness, stability, and communication efficiency under dynamic network disturbances.

## 6. Conclusions

This paper addresses the challenges posed by dynamic communication topologies and resource heterogeneity in distributed task allocation for multi-UAV systems and proposes a novel Two-Level Clustered CBBA (TLC-CBBA). By combining hierarchical clustering with a consensus-based task allocation mechanism, the proposed method achieves efficient, robust, and scalable task allocation in complex scenarios. In the first-layer clustering, UAVs are grouped based on network centrality measures to identify key communication nodes, thereby reducing redundant inter-group communication and enhancing the robustness of the network topology. In the second-layer clustering, a resource-balanced, distance-aware K-medoids algorithm is applied within each subgroup to further refine the clusters. This ensures spatial compactness while maintaining balanced resource distribution, thereby improving intra-cluster coordination and task compatibility. Within this two-level structure, each subgroup executes CBBA for local task bundling and consensus, while a lightweight inter-cluster coordination mechanism guarantees globally consistent and conflict-free task allocation. Simulation results indicate that TLC-CBBA significantly outperforms standard CBBA and its variants (e.g., DMCHBA, G-CBBA, and Clustering-CBBA) in terms of communication efficiency, overall task performance, and computational time, while maintaining stable performance across varying network densities and task scales. These findings validate the effectiveness and robustness of the proposed approach in dynamic and heterogeneous multi-UAV systems.

Future work will verify the proposed method in larger-scale UAV swarms and more complex task-coupling scenarios, while addressing practical issues such as computation delays, communication robustness, and performance under bandwidth constraints. Furthermore, the TLC-CBBA will be extended to an adaptive key-node architecture that adjusts key-node numbers based on network density and communication limits, enhancing scalability and real-world applicability in both military and civilian domains. 

## Figures and Tables

**Table 1:** Selection of key nodes in typical communication topology.

Typical Communication Topology	2 Key Nodes	3 Key Nodes	4 Key Nodes	5 Key Nodes
Fully connected	2, 24	2, 24, 15	2, 24, 16, 8	2, 24, 13, 16, 8
Dense	2, 24	2, 24, 14	2, 24, 14, 8	2, 24, 12, 14, 8
Star	0, 24	0, 23, 15	0, 24, 16, 8	0, 24, 11, 16, 8
Tree	2, 22	2, 24, 15	2, 24, 15, 9	2, 24, 13, 15, 9
Ring	2, 24	2, 24, 18	2, 24, 18, 8	2, 24, 15, 18, 8
Random	2, 24	2, 24, 14	2, 24, 14, 9	2, 24, 11, 14, 9

**Table 2:** UAVs and resource distribution based on K-medoids clustering algorithm with distance and resource balance.

Cluster $\left(K_{i}\right)$	UAVs $\left(U_{i}\right)$	Total Resources [Payload, Fuel, Capacity]
$K_{1}$	$\left\{U_{0},U_{1},U_{2},U_{17},U_{18},U_{19},U_{9},U_{10},U_{11}\right\}$	[69,1530,66]
$K_{2}$	$\left\{U_{3},U_{4},U_{5},U_{20},U_{21},U_{22},U_{12},U_{13},U_{14}\right\}$	[168,2700,102]
$K_{3}$	$\left\{U_{6},U_{7},U_{8},U_{23},U_{24},U_{15},U_{16}\right\}$	[257,2950,101]

**Table 3:** UAVs and resource distribution based on K-medoids clustering algorithm with distance.

Cluster $\left(K_{i}\right)$	UAVs $\left(U_{i}\right)$	Total Resources [Payload, Fuel, Capacity]
$K_{1}$	$\left\{U_{4},U_{5},U_{6},U_{7},U_{8}\right\}$	[350,2000,55]
$K_{2}$	$\left\{U_{12},U_{13},U_{14},U_{15},U_{16},U_{17},U_{18},U_{19},U_{20},U_{21},U_{22},U_{23},U_{24}\right\}$	[44,3730,152]
$K_{3}$	$\left\{U_{0},U_{1},U_{2},U_{3},U_{9},U_{10},U_{11}\right\}$	[100,1450,62]

**Table 4:** Statisticalevaluation and comparison of total task scores of different algorithms across 24 UAVs.

Algorithms	Average	Best	Worst	CPU Time	Confidence Interval
CBBA [36]	265	280	240	4.8	[215, 315]
DMCHBA [27]	300	310	289	3.8	[258, 342]
G-CBBA [44]	305	316	283	3.5	[270, 340]
Clustering-CBBA [8]	310	326	294	3.2	[280, 340]
TLC-CBBA	**403**	**430**	**388**	**1.5**	[**378**, **428**]

**Table 5:** Statistical comparison of communication steps across algorithms on 25 tasks.

Algorithms	Average	Best	Worst	CPU Time	Confidence Interval
CBBA [36]	4203	4002	4400	6.7	[4053, 4353]
DMCHBA [27]	1920	1745	2089	5.6	[1780, 2060]
G-CBBA [44]	1520	1347	1730	5.5	[1390, 1650]
Clustering-CBBA [8]	1245	1032	1417	4.2	[1125, 1365]
TLC-CBBA	**517**	**489**	**596**	**3.5**	[**419**, **615**]

**Table 6:** Effects of $w_{k}$, $\gamma_{1}$, and $\gamma_{2}$ on TLC–CBBA performance (30 tasks, 15 UAVs). Bold values denote the optimum results.

Parameter	Value	Average Total Score	Average Communication Steps	Runtime (s)
Variation of $w_{k}$ ($\gamma_{1}=\gamma_{2}=0.5$)				
$w_{k}$	0.10	364	610	5.1
	0.20	401	556	4.4
	**0.25**	**431**	**470**	**3.7**
	0.30	417	540	4.1
	0.40	406	617	4.2
	0.50	405	608	4.1
Variation of $\gamma_{1}$ ($w_{k}=0.25$, $\gamma_{2}=0.5$)				
$\gamma_{1}$	0.10	375	670	5.4
	0.30	411	590	4.2
	**0.50**	**429**	**479**	**3.9**
	0.70	401	520	4.7
	0.90	398	634	5.5
Variation of $\gamma_{2}$ ($w_{k}=0.25$, $\gamma_{1}=0.5$)				
$\gamma_{2}$	0.10	392	650	5.1
	0.30	410	532	4.2
	**0.50**	**425**	**483**	**3.8**
	0.70	399	592	4.5
	0.90	375	679	5.3

**Table 7:** Wilcoxon rank-sum test of runtime differences between TLC-CBBA and other algorithms.

Algorithm	*p*-Value	Symbol	Effect Size
TLC-CBBA–CBBA	2.926546 $\times 10^{-11}$	+	0.858540
TLC-CBBA–DMCHBA	2.902719 $\times 10^{-11}$	+	0.858695
TLC-CBBA–G-CBBA	2.891782 $\times 10^{-11}$	+	0.858767
TLC-CBBA–Clustering-CBBA	2.162663 $\times 10^{-6}$	+	0.611621

**Table 8:** Statistical Results of the Dynamic Online Evaluation.

Metric	Value	Unit
Number of re-clustering events	49	times
Average re-clustering delay	545.18	ms
Average number of control messages	346.24	messages/event
Average size of control data	279,782.69	bytes/event
Average UAV re-assignment ratio	0.855	–
Average task re-assignment ratio	0.374	–
Average waiting time	15.12	s

## Data Availability

All data supporting the findings of this study are contained within the article, which provides complete details of the simulation settings and parameters required for reproducibility. In addition, the corresponding source code has been made publicly available at https://github.com/ycchao0406/TLC_CBBA (accessed on 29 October 2025). Additional information can be obtained from the corresponding author upon reasonable request.
